# Diabetic Cardiovascular Autonomic Neuropathy Predicts Recurrent Cardiovascular Diseases in Patients with Type 2 Diabetes

**DOI:** 10.1371/journal.pone.0164807

**Published:** 2016-10-14

**Authors:** Seon-Ah Cha, Jae-Seung Yun, Tae-Seok Lim, Kyoungil Min, Ki-Ho Song, Ki-Dong Yoo, Yong-Moon Park, Yu-Bae Ahn, Seung-Hyun Ko

**Affiliations:** 1 Division of Endocrinology and Metabolism, Department of Internal Medicine, College of Medicine, The Catholic University of Korea, Seoul, Republic of Korea; 2 Division of Cardiology, Department of Internal Medicine, College of Medicine, The Catholic University of Korea, Seoul, Republic of Korea; 3 Epidemiology Branch, National Institute of Environmental Health Sciences, National Institutes of Health, Research Triangle Park, NC, United States of America; University of British Columbia, CANADA

## Abstract

Cardiovascular autonomic neuropathy (CAN) is a risk factor for cardiovascular disease (CVD) and mortality in patients with type 2 diabetes. This study evaluated the relationship between CAN and recurrent CVD in type 2 diabetes. A total of 206 patients with type 2 diabetes who had a history of CVD within 3 years of enrollment were consecutively recruited from January 2001 to December 2009 and followed-up until December 2015. Cardiovascular autonomic function tests were performed using the following heart rate variability parameters: expiration-to-inspiration ratio, response to Valsalva maneuver and standing. We estimated the recurrence of CVD events during the follow-up period. A total of 159 (77.2%) of the 206 patients enrolled completed the follow up, and 78 (49.1%) patients had recurrent episodes of CVD, with an incidence rate of 75.6 per 1,000 patient-years. The mean age and diabetes duration were 62.5 ± 8.7 and 9.2 ± 6.9 years, respectively. Patients who developed recurrent CVD also exhibited hypertension (*P* = 0.004), diabetic nephropathy (*P* = 0.012), higher mean systolic blood pressure (*P* = 0.006), urinary albumin excretion (*P* = 0.015), and mean triglyceride level (*P* = 0.035) than did patients without recurrent CVD. Multivariable Cox hazard regression analysis revealed that definite CAN was significantly associated with an increased risk of recurrent CVD (hazard ratio [HR] 3.03; 95% confidence interval [CI] 1.39−6.60; *P* = 0.005). Definite CAN was an independent predictor for recurrent CVD in patients with type 2 diabetes who had a known prior CVD event.

## Introduction

The worldwide prevalence of diabetes has increased gradually over the past several years [1]. The Korea National Health and Nutrition Examination Survey reported that the prevalence of diabetes increased from 8.6% in 2001 to 10.2% in 2014 [2]. Increased numbers of patients with type 2 diabetes are inevitably accompanied by diabetes-associated chronic vascular complications. The incidence of diabetes-related complications decreased over the past two decades in U.S., following improvements in glycemic control, acute clinical care, patient education, and advances in health care systems, but a large burden persists because of the continued increase in the number of patients with diabetes [3]. Moreover, obesity, hypertension, and dyslipidemia, known risk factors for cardiovascular disease (CVD), are also more frequent in subjects with diabetes than they are in the non-diabetic population [4–6]. The 2015 Korean Diabetes Fact Sheet reported that the incidence of coronary heart disease (CHD), and stroke was approximately four times and two times higher compared to the non-diabetic population, respectively [7]. Consequently, CVD is a major cause of death in patients with diabetes in Korea [8].

Multifactorial interventions, such as control of glycemia, blood pressure (BP), and dyslipidemia, are essential to prevent diabetic complications [9]. However, CVD prevention remains difficult in subjects with type 2 diabetes [10]. The International Clinical Practice Guidelines emphasize the importance of total diabetes care, but the percentage of patients who successfully manage their BP, blood glucose, and weight is quite low [11]. In Korea, only 14.5% of patients with type 2 diabetes reached the target range for BP, glucose, and lipid level [12]. Thus, considerable numbers of patients with type 2 diabetes are exposed to risks of CVD and CVD death, and CVD prevention remains difficult in subjects with type 2 diabetes in real practice [10–12]. Therefore, additional predictors or markers for CVD are needed for early detection and prevention of CVD in type 2 diabetes patients.

Cardiovascular autonomic neuropathy (CAN) manifests as a group of symptoms and signs, including exercise intolerance, resting tachycardia, and orthostatic or postural hypotension, that are common but insidious complications in diabetes [13–15]. The Detection of Ischemia in Asymptomatic Diabetics (DIAD) study demonstrated that CAN was an independent predictor for silent myocardial ischemia in subjects with type 2 diabetes [16, 17].

A meta-analysis of 12 cross-sectional studies revealed a significantly increased risk of silent myocardial ischemia in subjects with CAN compared to subjects without CAN, and the pooled prevalence rate risk for silent myocardial ischemia was 1.96 [14].

The risk of recurrent CVD was higher and more fatal outcomes were observed in patients with type 2 diabetes compared to non-diabetic subjects [18, 19]. The Secondary Analysis of the Stroke Prevention by Aggressive Reduction in Cholesterol Levels (SPARCL) Trial demonstrated that subjects with type 2 diabetes who had a history of ischemic or hemorrhagic stroke or transient ischemic attack exhibited a 1.6-fold higher risk of recurrent CVD [18]. The development of recurrent CVD is also a predictor for disability and cardiovascular mortality in type 2 diabetes patients [20]. However, the impact of CAN on the development of recurrent CVD in subjects with type 2 diabetes was rarely investigated.

This study estimated the association between CAN and recurrent CVD in patients with type 2 diabetes with a known prior CVD event. To the best of our knowledge, this study is the first report of the relationship between CAN and recurrent CVD in patients with type 2 diabetes.

## Materials and Methods

A total of 228 patients with type 2 diabetes, aged 25−75 years, who were diagnosed with a first-time CVD within the previous 3 years, were consecutively enrolled at the university-affiliated diabetes center of St. Vincent’s Hospital in South Korea from January 2001 to December 2009. CVD was defined using a diagnosed history of CHD (e.g., angina pectoris, nonfatal myocardial infarction [MI], or coronary revascularization, including percutaneous coronary intervention or coronary bypass surgery) or ischemic stroke [19, 21]. Stroke history was the composite history of transient ischemic attack or ischemic stroke [21]. Twenty-two patients with arrhythmia, type 1 diabetes, history of ketoacidosis or any severe illness, such as liver cirrhosis, heart failure, severe infection, end-stage renal disease, recent CVD within 6 months, or malignancy were excluded. The Catholic Medical Center Ethics Committee approved this study (approval number of Institutional Review Board [IRB]: VC10OISE0152). All participants provided their signed informed consent.

As described in detail previously, all subjects completed a standard questionnaire at baseline to obtain information on the subject’s past medical history, alcohol consumption status, current or past cigarette smoking status, and medication use. Hypertension was defined as systolic BP ≥ 140 mmHg, diastolic BP ≥ 90 mmHg, or the use of antihypertensive medications [22]. Subjects were classified as current smokers, past smokers, or non-smokers based on smoking status from the questionnaire. We defined current smoking as individuals who had smoked any tobacco products within the previous 12 months in the study [23]. Alcohol consumption was defined as drinking one to two drinks per day for six months or longer [24].

Laboratory data, including a lipid parameter comprised of total cholesterol, triglyceride, high-density lipoprotein cholesterol, and low-density lipoprotein cholesterol, fasting plasma glucose, and hemoglobin A1c (HbA1c) levels, were measured at baseline and every 6 months during the follow-up period. Fasting plasma glucose (FPG) and the lipid profile were assessed using an automated enzymatic method (736−40; Hitachi, Tokyo, Japan), and HbA1c was measured using high-performance liquid chromatography (Bio-Rad, Montreal, QC, Canada) [22]. Estimated glomerular filtration rate (eGFR) was assessed using the 4-component Modification of Diet in Renal Disease equation [25].

Diabetic retinopathy was assessed via a comprehensive eye examination by an ophthalmologist from retinal photographs taken at baseline. The urinary albumin excretion rates were assessed from a 24-hour urine collection or single-void urine specimens using immunoturbidimetry (Eiken, Tokyo, Japan). Diabetic nephropathy was defined as a urine albumin excretion (UAE) rate > 30 mg/day or urine albumin-to-creatinine ratio > 30 mg/g of creatinine in spot urine specimens, which was confirmed at least three times in six months [22, 26].

Participants received follow-up care every 3 to 4 months for usual diabetes care on an outpatient basis from enrollment to December 2015. BP was measured three times at each visit using a mercury sphygmomanometer (YAMASU, Saitama, Japan) after participants were seated for five minutes. The average of the three BP measurements was used in this study.

### Baseline cardiovascular autonomic function test

All participants were instructed to fast for 12 hours and to avoid nicotine, alcohol, insulin, diuretics, antidepressants, antihistamines, or sympatholytic drugs. A single skilled examiner performed cardiovascular autonomic function test (AFT) was performed according to the Ewing method [27]. The cardiovascular AFT included tests of heart rate variability, such as the expiration-to-inspiration (E/I) ratio, responses to the Valsalva maneuver, and postural changes from lying to standing [13, 14]. Each measurement was scored as normal = 0 or abnormal = 1, which was assessed by automated means using Monitor one nDx (QMed, Inc. Eatontown, NJ) and the total score was calculated with a total maximum score of 3 [13]. The result of each CAN item was reviewed by an investigator. CAN staging was determined from the total score of heart rate variability as follows: normal autonomic function = 0; early CAN = 1; and definite CAN ≥ 2 [15, 21]. Orthostatic hypotension was defined as a fall in BP >20 mmHg in systolic BP or >10 mmHg in diastolic BP in response to postural change from supine to standing [14].

### Evaluation of recurrent CVD

The primary endpoint of this study was a recurrent attack of CVD, which was defined as CHD, stroke or limb amputation from diabetic foot, according to World Health Organization (WHO) criteria [28–30]. CHD included MI, non-MI acute coronary syndrome, heart failure, or death attributable to CHD [28]. MI was defined as one of the following criteria: detection of a rise and/or fall of cardiac biomarkers with at least one marker of clinical cardiac ischemia and the absence of non-cardiac causes of biomarker elevation and cardiac symptoms or signs or electrocardiography using WHO criteria [30]. Stroke was defined as a neurological deficit due to cerebrovascular causes that persisted beyond 24 hours or led to death within 24 hours [21, 31]. Recurrence was diagnosed if CVD occurred at least 28 days after the prior event [29]. A physician evaluated whether the subjects had experienced recurrent CVD events based on the above-listed criteria or information from medical records if the subject visited another hospital for a recurrent CVD events, and specialists, including cardiologists, neurologists, or neurosurgeons, confirmed the clinical diagnosis of CVD based on verified medical records or clinical manifestations.

### Statistical analysis

All data are expressed as the means (standard deviation) or frequencies or medians with an interquartile range. *P* < 0.05 was considered significant. The Chi-square test was used to determine differences in the proportion of categorical variables, and independent Student’s *t*-tests evaluated differences between the means of two continuous variables. The Mann-Whitney test was used for non-normally distributed variables. Incidence rates were estimated using the person-year method and adjusted for age using the direct method. The proportionality assumption was examined using log-minus log-survival plots, and Cox proportional hazards regression was used to identify associations between CAN and recurrent CVD. The log-rank (Mantel-Cox) test was used to distinguish the effect of the three stages of CAN on recurrent CVD.

The association between CAN and recurrent CVD was analyzed after adjustment for the following factors: age, sex, diabetes duration, presence of hypertension, mean systolic BP, eGFR, and mean HbA1c during the follow-up period in the study. The results are presented as HRs and 95% CIs. Statistical analyzes were performed using SAS version 9.3 (SAS Institute, Cary, NC, USA).

## Results

Twenty-two patients were excluded, and 206 subjects with type 2 diabetes who had a prior CVD event within the previous 3 years were recruited. A total of 159 (77.2%) subjects completed the follow-up evaluation (S1 Fig). Forty-seven patients (22.8%) who dropped out or died of non-cardiovascular causes during the follow-up period from enrollment to December 2015 were excluded from the analysis (S1 Fig). The group of subjects who did not complete the follow-up were not significantly different in sex ratio (*P* = 0.379) or mean HbA1c (*P* = 0.208), but these subjects were older (65.9 ± 8.0 vs. 62.5 ± 8.7 years, *P* = 0.016) compared to the 159 subjects who completed the follow-up. The median follow-up period was 8.9 (7.5–10.8) years, and 17 patients (10.7%) died.

Mean age and diabetes duration at baseline were 62.5 ± 8.7 and 9.2 ± 6.9 years, respectively, and 59.1% of the subjects were women in this study. A total of 68.6% patients had hypertension, and the baseline HbA1c level was 8.9% (Table 1).

**Table 1:** Comparison of characteristics of participants with and without recurrent cardiovascular events.

Characteristic	Total	Recurrent cardiovascular events		*P* value
		yes (n = 78)	no (n = 81)	
Age (years)	62.5 ± 8.7	61.9 ± 9.0	63.0 ± 8.4	0.545
Female sex (%)	59.1	56.4	61.7	0.495
Diabetes duration (years)	9.2 ± 6.9	9.6 ± 7.2	8.8 ± 6.6	0.599
BMI (kg/m^2^)	25.3 ± 3.3	25.1± 3.5	25.4 ± 3.0	0.617
Hypertension (%)	68.6	79.5	58.0	0.004
Alcohol use (%)	20.1	21.8	18.5	0.606
Smoking (%)	29.6	30.8	28.4	0.743
Diabetic retinopathy (%)	44.1	50.7	38.2	0.130
Diabetic nephropathy (%)	34.0	43.6	24.7	0.012
SBP (mmHg)	124.1 ± 19.8	127.0 ± 18.0	121.7 ± 20.9	0.092
DBP (mmHg)	73.6 ± 12.1	75.0 ± 9.9	72.4 ± 13.7	0.201
**Treatment (%)**				
Insulin	40.9	47.4	34.6	0.099
Sulfonylurea	54.7	51.3	58.0	0.393
Metformin	30.8	34.6	27.2	0.309
ACE inhibitors or ARBs	43.4	50.0	37.0	0.099
β-blocker	11.3	14.1	8.6	0.277
Antiplatelet agents	78.6	78.2	79.0	0.901
Statin	17.0	16.7	17.3	0.495
**Laboratory test at baseline**				
FPG (mmol/L)	9.87 ± 4.51	9.63 ± 3.67	10.09 ± 5.19	0.783
eGFR (mL/min/1.73 m^2^)	83.1 ± 18.0	82.6 ± 18.5	83.6 ± 17.6	0.710
TC (mmol/L)	4.75 ± 0.95	4.73 ± 1.01	4.78 ± 0.89	0.733
TG (mmol/L)	1.53 (1.15–2.19)	1.68 (1.21–2.31)	1.43 (1.10–1.97)	0.072
HDL-C (mmol/L)	1.07 ± 0.30	1.03 ± 0.28	1.10 ± 0.31	0.147
LDL-C (mmol/L)	2.85 ± 0.85	2.84 ± 0.91	2.86 ± 0.79	0.906
Baseline HbA1c (mmol/mol)	73.9 ± 23.0	72.4 ± 21.6	75.4 ± 24.2	0.722
Urinary albumin excretion (mg/day)	13.0 (7.0–54.9)	18.0 (9.2–140.9)	11.2 (5.9–38.8)	0.015
**Blood pressure and laboratory test at follow-up**				
Mean SBP (mmHg)	128.2 ± 10.4	130.3 ± 11.1	126.2 ± 9.4	0.006
Mean DBP (mmHg)	71.5 ± 7.5	72.3 ± 8.2	70.7 ± 6.7	0.275
Mean HbA1c (mmol/mol)	68.2 ± 15.3	69.0 ± 14.1	67.3 ± 16.5	0.447
Mean TC (mmol/L)	4.47 ± 0.65	4.49 ± 0.62	4.45 ± 0.68	0.690
Mean TG (mmol/L)	1.50 (1.24–2.02)	1.68 (1.32–2.10)	1.38 (1.05–1.93)	0.035
Mean HDL-C (mmol/L)	1.04 ± 0.24	1.02 ± 0.27	1.07 ± 0.21	0.089
Mean LDL-C (mmol/L)	2.60 ± 0.59	2.60 ± 0.61	2.59 ± 0.58	0.858

Values are presented as the means ± standard deviation or percentage or medians (interquartile range).

SBP, systolic blood pressure; DBP, diastolic blood pressure; ACE inhibitor, angiotensin-converting enzyme inhibitor; ARB, angiotensin receptor blocker; FPG, fasting plasma glucose; eGFR, estimated glomerular filtration rate; TC, total cholesterol; TG, triglyceride; IQR, interquartile range; HDL-C, high-density lipoprotein cholesterol; LDL-C, low-density lipoprotein cholesterol.

A total of 78 patients (49.1%) experienced recurrent CVD during the follow-up period, which resulted in an incidence rate of 75.6 per 1,000 patient-years. The median times from the prior cardiovascular event to the time of AFT and recurrent cardiovascular events were 18.0 (11.0–24.0) months and 36.0 (24.0–72.0) months, respectively. The numbers of patients with recurrent ischemic stroke, CHD, and limb amputations were 29, 47 and 2, respectively. S1 Table describes the prior and recurrent CVDs.

Table 1 described the baseline characteristics of participants in the recurrent CVD group and without recurrent CVD group. Patients who developed recurrent CVD exhibited more hypertension (*P* = 0.004), diabetic nephropathy (*P* = 0.012), higher mean systolic BP (*P* = 0.006), urinary albumin excretion (*P* = 0.015), and mean triglyceride level (*P* = 0.035) compared to patients who did not have recurrent CVD (Table 1). However, there were no significant differences in the diabetes duration, sex ratio, eGFR, mean HbA1c, insulin use, and mean LDL or HDL cholesterol levels between the groups.

Table 2 showed baseline cardiovascular AFT results according to recurrent CVD. At baseline, 43.4% of patients had definite CAN and 35.9% of patients had orthostatic hypotension. Patients with recurrent CVD were more likely to exhibit an abnormal Valsalva ratio (*P* = 0.004) and posture ratio (*P* = 0.014) compared to patients without recurrent CVD. The stage of CAN was also higher in patients with recurrent CVD (*P* = 0.005), but an abnormal E/I ratio (*P* = 0.394) or the presence of orthostatic hypotension (*P* = 0.561) was not significantly different compared with patients without recurrent CVD (Table 2).

**Table 2:** Cardiovascular autonomic neuropathy and recurrent cardiovascular events.

	Total	Recurrent cardiovascular events		*P* value
		yes (n = 78)	no (n = 81)	
Abnormal E/I ratio	28.9	32.1	25.9	0.394
Abnormal valsalva ratio	60.4	71.8	49.4	0.004
Abnormal posture ratio	44.0	53.8	34.6	0.014
Staging of CAN				0.005
Normal	26.4	16.7	35.8	
Early	30.2	28.2	32.1	
Definite	43.4	55.1	32.1	
Orthostatic hypotension	35.9	38.5	33.8	0.561

Values are presented as %. E/I, expiration-to-inspiration. Orthostatic hypotension was defined as a fall in blood pressure > 20 mmHg in systolic blood pressure or > 10 mmHg in diastolic blood pressure in response to postural change from supine to standing.

CAN, cardiovascular autonomic neuropathy.

**Fig 1:**
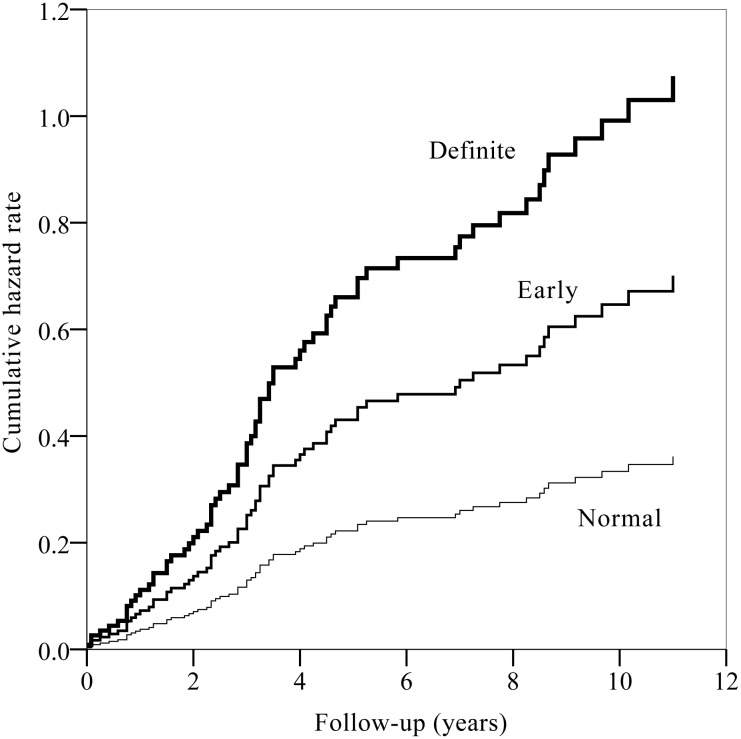
Cumulative hazard rate of recurrent cardiovascular diseases according to the stages of cardiovascular autonomic neuropathy in patients with type 2 diabetes. (log-rank *P* = 0.001).

Univariable Cox proportional hazard regression analysis revealed that patients with definite CAN exhibited a significantly higher risk of recurrent CVD ([hazard ratio] HR 2.90; 95% CI 1.56−5.40; *P* = 0.001). The presence of hypertension and mean systolic BP were also significant predictors for recurrent CVD in univariable analysis, but the presence of hypertension was not an independent risk factor of recurrent CVD after adjustment for multiple confounders (S2 Table). Multivariable Cox proportional hazard regression analysis revealed that patients with definite CAN exhibited a 3.0-fold higher risk of recurrent CVD than did patients with normal autonomic function after adjusting for age, sex, diabetes duration, presence of hypertension, mean systolic BP, eGFR, mean LDL cholesterol, and mean HbA1c (HR 3.03; 95% CI 1.39−6.60; *P* = 0.005) (Fig 1, Table 3, S2 Table).

**Table 3:** Multivariable Cox hazards regression model for the risk of recurrent cardiovascular diseases.

	Model 1	Model 2	Model 3	Model 4
Staging of CAN	HR (95% CI)	HR (95% CI)	HR (95% CI)	HR (95% CI)
Normal	1.00	1.00	1.00	1.00
Early	2.31 (1.05–5.06)^a^	2.16 (0.98–4.75)	1.87 (0.84–4.15)	1.93 (0.86–4.36)
Definite	3.56 (1.71–7.42)^a^	3.16 (1.51–6.63)^a^	2.74(1.29–5.86)^a^	3.03 (1.39–6.60)^a^

Multivariable Cox proportional hazard models were adjusted for the following covariates: model 1: sex, age and diabetes duration; model 2: model 1 + presence of hypertension; model 3: model 2 + mean SBP; and model 4: model 3 + eGFR, mean LDL-C and mean HbA1c.

^a^*P* < 0.05

CAN, cardiovascular autonomic neuropathy; HR, hazard ratio; CI, confidence interval; SBP, systolic blood pressure; eGFR, estimated glomerular filtration rate; LDL-C, low-density lipoprotein cholesterol.

Mean systolic BP was also a significant predictor for recurrent CVD compared to groups without recurrent CVD (HR 1.28; 95% CI 1.02−1.62; *P* = 0.037, S2 Table). Presence of hypertension and diabetic nephropathy were more frequent in groups with recurrent CVD, but these factors were not statistically significant in multivariable Cox proportional hazard regression model. Fig 1 showed the HR stratified by CAN stages at baseline in this study.

## Discussion

This prospective cohort study demonstrated that cardiovascular autonomic dysfunction was a risk factor for recurrent CVD in patients with type 2 diabetes. The recurrence of CVD was independent of age, sex, diabetes duration, presence of hypertension, mean systolic BP level, glycemic control status, and baseline renal function. In this population, 49.1% of patients with prior CVD developed recurrent CVDs during the 9-year follow-up period, which corresponds to the incidence rate of 75.6 per 1,000 patient-years and this value is comparable with that found in another prospective study [19]. Our study found that BP control status was also an important predictor for recurrent CVD in patients with type 2 diabetes. Seventeen patients died due to recurrent CVD during follow-up periods.

CAN is a clinically important diabetic autonomic neuropathy because of the increased risk of mortality in patients with diabetic CAN [32]. The prevalence of CAN varies from 12.2 to 22.1% in patients with type 2 diabetes, but it exhibits wide variation across study designs and populations [33]. Ewing et al. reported that patients with symptomatic CAN had a high mortality rate, with 50% of patients exhibiting with abnormal results and autonomic symptoms dying within 2.5 years [34]. A meta-analysis of 15 studies reported that CAN was a risk factor for mortality in diabetes with a pooled relative risk for all-cause mortality of 3.45 (95% CI 2.66–4.47; *P* = 0.001) [35]. The Action to Control Cardiovascular Risk in Diabetes (ACCORD) trial demonstrated that participants with baseline CAN, which was verified from the lowest quartile of heart rate variability, QT interval prolongation and resting heart rate, exhibited a 1.6–2.1 times higher risk of all-cause mortality and 1.9−2.6 times higher risk of cardiovascular mortality [36]. In addition, a second analysis of the DIAD study showed CAN was significantly associated with CHD and CV death [17].

The presence of CAN was associated with silent myocardial ischemia and major cardiac events in previous studies [14, 16, 17, 37]. Toyry et al. found that CAN was an independent risk factor for stroke in type 2 diabetes [38]. In addition, we also reported that CAN predicted acute ischemic stroke in patients with type 2 diabetes in a 7-year follow-up study [27].

Recurrent CVD has more fatal outcomes in patients with type 2 diabetes than those without diabetes [18]. CAN might contribute to the increase in mortality from recurrent CVD in patients with type 2 diabetes. However, few studies report the relationship between CAN and recurrent CVD. The presence of CAN may be a direct cause that leads to recurrent CVD, or it may result from previous CVD. Combined comorbidities, such as hypertension, metabolic syndrome, chronic kidney disease, and other diabetic microvascular complications, may contribute to the recurrence of a CVD attack and should be considered as risk factors [32].

A previous study demonstrated that the association between CAN and CHD remained significant in patients with diabetes after adjustment for silent myocardial ischemia ([odds ratio] OR 4.30; 95% CI 1.07−17.31; *P* = 0.04) [37]. We also adjusted for multiple confounders, including age, sex, the presence of hypertension, diabetes duration, renal function, and BP, glycemic and, lipid control status, during the observation period. This study demonstrated that CAN was an independent predictor for recurrent CVD in patients with type 2 diabetes after adjustment for metabolic and clinical factors. Therefore, patients with type 2 diabetes who experienced a previous CVD should be monitored for the presence of definite CAN, and attention to control other metabolic factors may be required to prevent recurrent CVDs.

The mechanisms by which CAN increases cardiovascular mortality and silent myocardial ischemia are not clear, but possible explanations for cardiovascular mortality include exercise intolerance, CHD risk, prolongation of the QT interval that was associated with lethal arrhythmia [14, 15]. CHD occurs from an imbalance between myocardial oxygen supply and demand [39]. Heart rate, myocardial contractility, afterload and preload influence myocardial oxygen demand [39]. The mechanism of silent myocardial ischemia may involve the inability to reach pain threshold during ischemia, a defective angina warning system, a higher beta-endorphin levels, and anti-inflammatory cytokines [40].

There were several limitations in our study. First, we have no normal values for the E/I ratio, Valsalva ratio, and posture ratio that are specific for Korean subjects or Korean patients with type 2 diabetes. However, one examiner performed AFT using the same method for the entire observation time and ethnic differences in AFT are not confirmed [41]. Second, 23.0% of the participants did not complete the follow up, which may result in selection bias in this study. Third, our study size was small. We confined patients with a prior CVD within 3 previous years of enrollment because of the possibility of the unexpected influence of prior CVD on cardiovascular autonomic function. Fourth, this study was an observational cohort design and only BP levels reached the target range during the study period. Mean LDL cholesterol and mean HbA1c levels were higher than American College of Cardiology and the American Heart Association (ACC/AHA) 2013 guidelines for the treatment of hypercholesterolemia [42].

This study has plausible strength despite these limitations. We observed the participants for 9 years with regular follow up of glucose, BP, and lipid profile status. This study has clinical implications that cardiovascular AFT may predict the recurrence of CVD in patients with type 2 diabetes independent of glycemic control, diabetes duration, age, sex, the presence of hypertension, mean BP, and kidney function.

In conclusion, this study suggests that definite CAN is an important predictable factor for the future development of recurrent CVD in patients with type 2 diabetes. Clinicians should closely monitor for the presence on cardiovascular autonomic dysfunction in type 2 diabetic patients with history of CVD for possible CVD recurrence. Further studies should be performed to investigate the pathogenic mechanism of CAN in the development of CVD.

## Supporting Information

S1 Fig Study flow chart. (PDF)

S1 Table Prior cardiovascular event and recurrent cardiovascular event in subjectswith recurrent cardiovascular diseases. Values are presented as n (%).CV, cardiovascular; CHD, coronary heart disease.
(DOCX)

S2 Table Univariable and multivariable Cox hazards regression model for the risk of recurrent cardiovascular diseases. eGFR, estimated glomerular filtration rate; TG, triglyceride; LDL-C, low-density lipoprotein cholesterol; SBP, systolic blood pressure; CAN, cardiovascular autonomic neuropathy.
(DOCX)
