# Supplementary figures and images for: Diabetic Cardiovascular Autonomic Neuropathy Predicts Recurrent Cardiovascular Diseases in Patients with Type 2 Diabetes
Source: PLoS One. 2016 Oct 14;11(10):e0164807. doi: 10.1371/journal.pone.0164807 (PMC5065186; doi:10.1371/journal.pone.0164807)

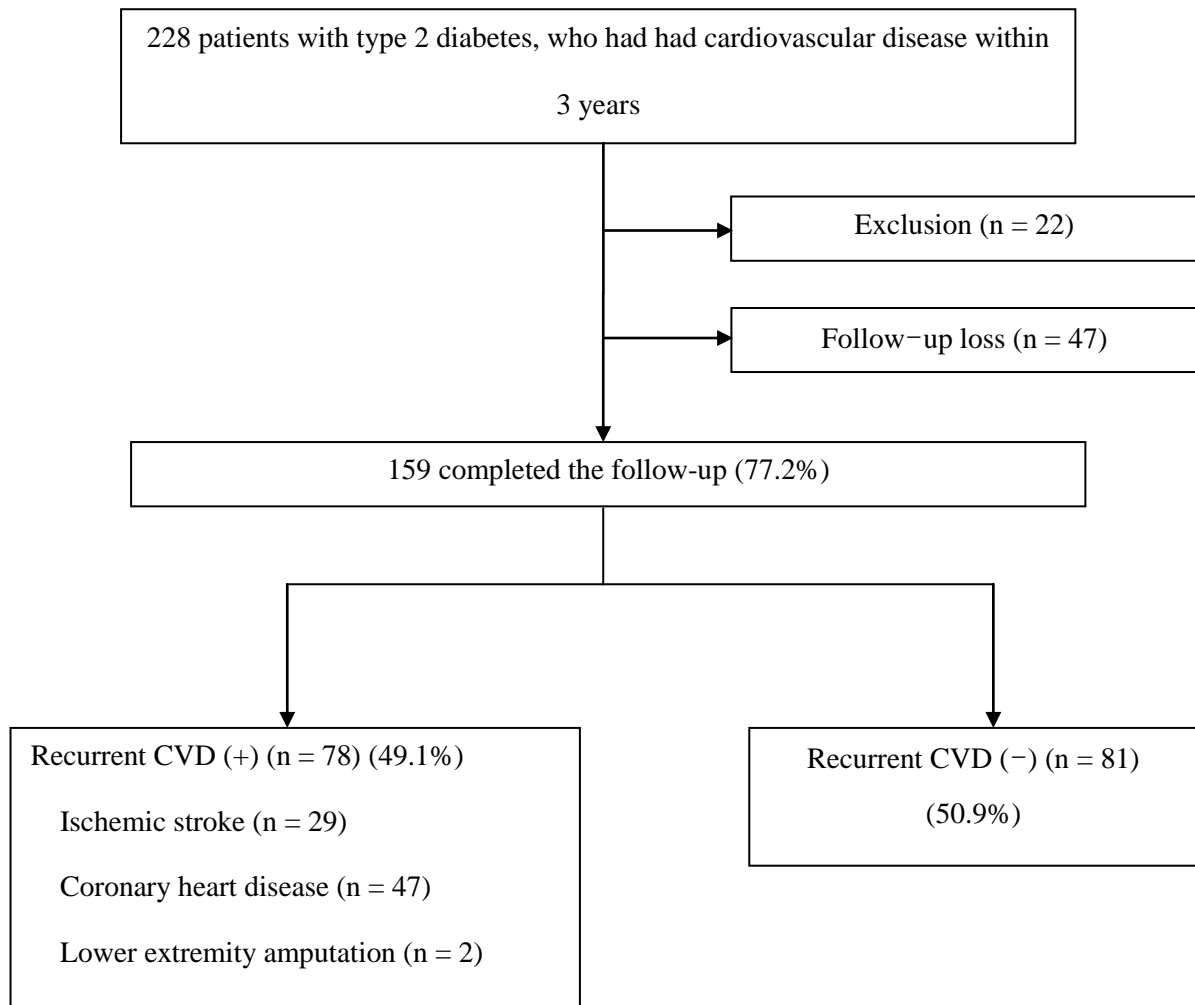

**S1 Fig. Study flow chart.**

Supplement: S1 Fig — (PDF) [file pone.0164807.s001.pdf]
