# Supplementary material for: Diabetic Cardiovascular Autonomic Neuropathy Predicts Recurrent Cardiovascular Diseases in Patients with Type 2 Diabetes
Source: PLoS One. 2016 Oct 14;11(10):e0164807. doi: 10.1371/journal.pone.0164807 (PMC5065186; doi:10.1371/journal.pone.0164807)
Supplement: S1 Table — Values are presented as n (%).CV, cardiovascular; CHD, coronary heart disease. (DOCX) [file pone.0164807.s002.docx]

**S1 Table. Prior cardiovascular event and recurrent cardiovascular event in subjects with recurrent cardiovascular diseases**

| Prior CV event | Recurrent CV event | n (%) | Prior CV event | Recurrent CV event | n (%) |
| --- | --- | --- | --- | --- | --- |
| Stroke  (n=43) | Stroke | 21 (26.9) | CHD  (n=35) | Stroke | 8 (10.3) |
|  | CHD | 21 (26.9) |  | CHD | 26 (33.3) |
|  | Limb amputation | 1 (1.3) |  | Limb amputation | 1 (1.3) |
