# Supplementary material for: Diabetic Cardiovascular Autonomic Neuropathy Predicts Recurrent Cardiovascular Diseases in Patients with Type 2 Diabetes
Source: PLoS One. 2016 Oct 14;11(10):e0164807. doi: 10.1371/journal.pone.0164807 (PMC5065186; doi:10.1371/journal.pone.0164807)
Supplement: S2 Table — eGFR, estimated glomerular filtration rate; TG, triglyceride; LDL-C, low-density lipoprotein cholesterol; SBP, systolic blood pressure; CAN, cardiovascular autonomic neuropathy. (DOCX) [file pone.0164807.s003.docx]

**S2 Table. Univariable and multivariable Cox hazards regression model for the risk of recurrent cardiovascular diseases**

|  | Unadjusted | | Adjusted | |
| --- | --- | --- | --- | --- |
|  | Hazard ratio (95% CI) | *P* value | Hazard ratio (95% CI) | *P* value |
| Age (per 10 years) | 0.78 (0.19–3.21) | 0.726 | 0.55(0.08–3.99) | 0.556 |
| Diabetes duration (per year) | 1.15 (0.85–1.56) | 0.359 | 1.01 (0.73–1.40) | 0.964 |
| Sex (women) | 0.90 (0.58–1.41) | 0.650 | 0.71 (0.42–1.18) | 0.182 |
| Hypertension (yes vs. no) | 2.23 (1.29–3.87) | 0.004 | 1.54 (0.85–2.79) | 0.156 |
| eGFR (mL/min/1.73 m^2^) | 0.67 (0.27–1.66) | 0.387 | 0.41 (0.13–1.28) | 0.124 |
| Mean TG (mg/dL) | 1.64 (0.97–2.78) | 0.068 | – |  |
| Mean LDL−C (mmol/L) | 1.17 (0.46–2.98) | 0.745 | 1.28 (0.47−3.52) | 0.627 |
| Mean SBP (per 10mmHg) | 1.45 (1.17−1.80) | 0.001 | 1.28 (1.02−1.62) | 0.037 |
| Mean HbA1c (mmol/mol) | 1.55 (0.41–5.89) | 0.518 | 1.06 (0.87–1.28) | 0.575 |
| Orthostatic hypotension | 1.17 (0.71–1.93) | 0.536 | – |  |
| Staging of CAN |  |  |  |  |
| Normal | 1.00 |  | 1.00 |  |
| Early | 1.73 (0.87–3.44) | 0.117 | 1.93 (0.86−4.36) | 0.113 |
| Definite | 2.90 (1.56–5.40) | 0.001 | 3.03 (1.39−6.60) | 0.005 |
